# Supplementary material for: Content-rich biological network constructed by mining PubMed abstracts
Source: BMC Bioinformatics. 2004 Oct 8;5:147. doi: 10.1186/1471-2105-5-147 (PMC528731; doi:10.1186/1471-2105-5-147)
Supplement: Additional File 2 — The original results of the above study (non-essential files are deleted to keep the file size under the limit set by BMC bioinformatics). [file 1471-2105-5-147-S2.bz2 › chilibotAdditionalFile2/dip50/583ID9533885E10951/html/NUTF2_RAN.html]

 


 **NUTF2** and **RAN** 
  
Found 47 abstracts in PubMed, retrieved 47.  
 

 What does Google say? 
 PDF only 
| .edu only 

---

**Interactive relationship** (e.g. stimulation, inhibition, etc)

**Stimulatory relationship**- An NTF2  [ **NUTF2** ]  mutant that cannot bind  **Ran**  6 was unable to facilitate  **Ran**  import.  Ref: 9889103 Curr Biol< MedlineTA>Curr Biol,
- Recently, it has been reported that NTF2  [ **NUTF2** ]  can stimulate the accumulation of  **Ran**  in digitonin permeabilized cells.  Ref: 10679025 Mol Biol Cell, 2000
- Monoclonal antibodies to NTF2  [ **NUTF2** ]  inhibit nuclear protein import by preventing nuclear translocation of the GTPase  **Ran** .  Ref: 10679025 Mol Biol Cell, 2000
- These results indicate that, instead,  **Ran**  increases the binding of NTF2  [ **NUTF2** ]  to nucleoporins by another mechanism, most probably by  **Ran**  itself binding to nucleoporins and NTF2  [ **NUTF2** ]  binding to this nuclear pore associated  **Ran** .  Ref: 10681579 J Biol Chem, 2000
- In this system, export is dependent on exogenous CRM1 Exportin1 a nuclear export receptor the GTPase  **Ran**  and nucleotide triphosphates NTPs, and is further stimulated by  **Ran**  binding protein 1 RanBP1 and nuclear transport factor 2  [ **NUTF2** ]  NTF2  [ **NUTF2** ] .  Ref: 9889120 Curr Biol, 1999
- Moreover, anti NTF2  [ **NUTF2** ]  mAbs inhibited nuclear import of both  **Ran**  and NLS containing protein in vitro, suggesting that NTF2  [ **NUTF2** ]  stimulates NLS dependent protein import by driving the nuclear accumulation of  **Ran** .  Ref: 10679025 Mol Biol Cell, 2000
- Nuclear transport factor 2  [ **NUTF2** ]  NTF2  [ **NUTF2** ]  facilitates protein transport into the nucleus and interacts with both the small Ras like GTPase  **Ran**  and nucleoporin p62.  Ref: 8757804 J Mol Biol, 1996
**Neutral relationship**- The interaction interface involves primarily the putative switch II loop of  **Ran**  residues 65 to 78 and the hydrophobic cavity and surrounding surface of NTF2  [ **NUTF2** ] .  Ref: 9533885 J Mol Biol, 1998
- While transport factors such as  **Ran**  and NTF2  [ **NUTF2** ]  show only transient interaction with the pores, a number of soluble transport receptors, including importin beta, show a tight association with the purified pores.  Ref: 11208084 Traffic, 2000
- The interaction between  **Ran**  and NTF2  [ **NUTF2** ]  is required for cell cycle progression.  Ref: 10930458 Mol Biol Cell, 2000
- NTF2  [ **NUTF2** ]  binds GDP  **Ran**  selectively and this interaction is important for efficient nuclear protein import in vivo.  Ref: 9533885 J Mol Biol, 1998
- NTF2  [ **NUTF2** ]  mediates nuclear import of  **Ran** .  Ref: 9822603 EMBO J, 1998
- We demonstrate that nuclear transport factor 2  [ **NUTF2** ]  NTF2  [ **NUTF2** ] , nucleoporin p62 and the Ras like GTPase  **Ran**  bind to the importin heterodimer via its beta subunit.  Ref: 9102465 J Mol Biol, 1997
- This provides the first evidence that NTF2  [ **NUTF2** ]  regulates the distribution of  **Ran**  in vivo.  Ref: 10679025 Mol Biol Cell, 2000
- These findings demonstrate that NTF2  [ **NUTF2** ]  is required to transport  **Ran**  into the nucleus in vivo.  Ref: 10930458 Mol Biol Cell, 2000
- Identification of an NTF2  [ **NUTF2** ]  related factor that binds  **Ran**  GTP and regulates nuclear protein export.  Ref: 10567585 Mol Cell Biol, 1999
- A temperature sensitive ts mutant of Saccharomyces cerevisiae NTF2  [ **NUTF2** ]  that does not bind to  **Ran**  is unable to import  **Ran**  into the nucleus at the nonpermissive temperature.  Ref: 10930458 Mol Biol Cell, 2000
- We conclude that interaction between NTF2  [ **NUTF2** ]  and GDP  **Ran**  is important for efficient nuclear protein import.  Ref: 9368653 J Mol Biol, 1997
- The interaction between NTF2  [ **NUTF2** ]  and  **Ran**  is essential for the import of  **Ran**  into the nucleus.  Ref: 11489893 J Biol Chem, 2001
- A GTP bound form of a  **Ran**  mutant that cannot bind NTF2  [ **NUTF2** ]  was not a substrate for import.  Ref: 9889103 Curr Biol< MedlineTA>Curr Biol, 2001
- It has been reported that p10 NTF2  [ **NUTF2** ]  interacts directly and specifically with  **Ran**  GDP but not with  **Ran**  GTP 6.  Ref: 9843686 Curr Biol, 1998
- Nuclear import of  **Ran**  is mediated by the transport factor NTF2  [ **NUTF2** ] .  Ref: 9889103 Curr Biol< MedlineTA>Curr Biol, 1998
- Nucleotide specific interaction of  **Ran**  TC4  [ **RAN** ]  with nuclear transport factors NTF2  [ **NUTF2** ]  and p97.  Ref: 8755535 Proc Natl Acad Sci U S A, 1996
- These interactions could help target  **Ran**  and NTF2  [ **NUTF2** ]  to a series of putative docking sites for the importin substrate complex located along the central transport channel of the NPC and so facilitate the passage of import material being transported from the cytoplasm into the nucleus.  Ref: 8918934 J Mol Biol, 1996
- Here we show that cytoplasmic injection of anti NTF2  [ **NUTF2** ]  mAbs resulted in a dramatic relocalization of  **Ran**  to the cytoplasm.  Ref: 10679025 Mol Biol Cell, 2000
- NTF2  [ **NUTF2** ]  may serve to modulate a transport step involving  **Ran**  TC4  [ **RAN** ] .  Ref: 9261173 J Biol Chem, 1997
- The addition of energy and nuclear transport factor 2  [ **NUTF2** ]  NTF2  [ **NUTF2** ]  5 was sufficient for the accumulation of  **Ran**  in the nucleus.  Ref: 9889103 Curr Biol< MedlineTA>Curr Biol, 1997
- We show that nuclear transport factor 2  [ **NUTF2** ]  NTF2  [ **NUTF2** ]  is required to concentrate  **Ran**  in the nucleus in the budding yeast, Saccharomyces cerevisiae.  Ref: 10889207 J Biol Chem, 2000
- A direct NTF2  [ **NUTF2** ]  RanGDP interaction is crucial for this process, since point mutations that disturb the RanGDP NTF2  [ **NUTF2** ]  interaction also interfere with  **Ran**  import.  Ref: 9822603 EMBO J, 1998
- NTF2  [ **NUTF2** ]  mediated import of  **Ran**  appears to be the bottleneck for maximal capacity of  **Ran**  driven nuclear transport.  Ref: 12606574 EMBO J, 2003
- Evidence that NTF2  [ **NUTF2** ]  directly mediates  **Ran**  import or that NTF2  [ **NUTF2** ]  is required to maintain the nuclear concentration of  **Ran**  in living cells has not been obtained.  Ref: 10679025 Mol Biol Cell, 2000
- Taken together, our data show that NTF2  [ **NUTF2** ]  is an essential regulator of the  **Ran**  distribution in living cells.  Ref: 10679025 Mol Biol Cell, 2000
- This interaction between NTF2  [ **NUTF2** ]  and GDP  **Ran**  is required for efficient nuclear protein import and may function between the docking and translocation steps of the pathway.  Ref: 9878368 J Mol Biol, 1998
- We used the crystal structure of the GDPRan NTF2  [ **NUTF2** ]  complex to design mutants in the switch II loop of  **Ran**  to probe the contribution of Lys71, Phe72 and Arg76 to this interaction.  Ref: 10356329 J Mol Biol, 1999
- Nuclear protein import requires a precisely choreographed series of interactions between nuclear pore components and soluble factors such as importin beta,  **Ran** , and nuclear transport factor 2  [ **NUTF2** ]  NTF2  [ **NUTF2** ] .  Ref: 10356329 J Mol Biol, 1999
- Previous biochemical studies have shown that NTF2  [ **NUTF2** ]  binds directly to the GDP bound form of  **Ran**  TC4  [ **RAN** ]  and to proteins of the nuclear pore complex that contain phenylalanine glycine repeats.  Ref: 9261173 J Biol Chem, 1997
- Structural basis for molecular recognition between nuclear transport factor 2  [ **NUTF2** ]  NTF2  [ **NUTF2** ]  and the GDP bound form of the Ras family GTPase  **Ran** .  Ref: 9533885 J Mol Biol, 1998
- The major contribution to the interaction made by the switch II loop accounts for the ability of NTF2  [ **NUTF2** ]  to discriminate between GDP and GTP bound forms of  **Ran** .  Ref: 9533885 J Mol Biol, 1998
- Furthermore, we found that high levels of  **Ran**  TC4  [ **RAN** ]  can relieve the requirement for NTF2  [ **NUTF2** ]  in a mammalian permeabilized cell assay for nuclear protein import.  Ref: 9261173 J Biol Chem, 1997
- **Ran**  function is modulated in a noncatalytic fashion by NTF2  [ **NUTF2** ] , a protein that mediates nuclear import of  **Ran**  GDP.  Ref: 10567585 Mol Cell Biol, 1999
- T42A  **Ran** .GDP also retains the ability to bind p10 NTF2  [ **NUTF2** ] , a component of the nuclear import pathway.  Ref: 9398678 Mol Biol Cell, 1997
- Furthermore, when the NTF2  [ **NUTF2** ]  E42K and D92N D94N NTF2  [ **NUTF2** ]  mutants that failed to bind GDP  **Ran**  in vitro were substituted for the chromosomal yeast NTF2  [ **NUTF2** ] , the yeast cells became non viable.  Ref: 9368653 J Mol Biol, 1997
- NTF2  [ **NUTF2** ]  has been shown to bind nuclear pore complex proteins and the GDP form of  **Ran**  in vitro.  Ref: 10679025 Mol Biol Cell, 2000
- Because the interaction interface maintains the positions of key  **Ran**  residues involved in binding MgGDP, NTF2  [ **NUTF2** ]  binding may help stabilize the switch state of  **Ran** , possibly in the context of targeting it to other components of the nuclear protein import machinery to specify directionality of transport.  Ref: 9533885 J Mol Biol, 1998
- Here we describe the results of solution and solid phase binding assays, which demonstrate that the small GTPase  **Ran**  TC4  [ **RAN** ]  interacts directly with the cytosolic transport factors p97 and NTF2  [ **NUTF2** ] .  Ref: 8755535 Proc Natl Acad Sci U S A, 1996
- Here we characterize a novel component of the  **Ran**  system that is 26% identical to NTF2  [ **NUTF2** ] , which based on its function we refer to as NTF2  [ **NUTF2** ]  related export protein 1 NXT1.  Ref: 10567585 Mol Cell Biol, 1999
- Engineered mutants in the switch II loop of  **Ran**  define the contribution made by key residues to the interaction with nuclear transport factor 2  [ **NUTF2** ]  NTF2  [ **NUTF2** ]  and the role of this interaction in nuclear protein import.  Ref: 10356329 J Mol Biol, 1999
- The release of  **Ran**  from NTF2  [ **NUTF2** ]  into the nucleus, and thus the directionality of  **Ran**  import, probably involves nucleotide exchange to generate RanGTP, for which NTF2  [ **NUTF2** ]  has no detectable affinity, followed by binding of the RanGTP to an importin beta family transport receptor.  Ref: 9822603 EMBO J, 1998
- These salt bridges account for the inhibition of the  **Ran**  NTF2  [ **NUTF2** ]  interaction by NTF2  [ **NUTF2** ]  mutants such as E42 K and D92 94N in which the negatively charged residues surrounding the cavity were altered.  Ref: 9533885 J Mol Biol, 1998
- The NTF2  [ **NUTF2** ]  hydrophobic cavity is a candidate for a potential binding site for other proteins involved in nuclear import such as  **Ran**  and nucleoporin p62.  Ref: 8757804 J Mol Biol, 1996
- Proteins that regulate the GTPase cycle and subcellular distribution of  **Ran**  include the cytoplasmic GTPase activating protein RanGAP and its co factors RanBP1, RanBP2, the nuclear guanine nucleotide exchange factor RanGEF, and the  **Ran**  import receptor NTF2  [ **NUTF2** ] .  Ref: 10811801 J Biol Chem, 2000
- Bacterially expressed rat NTF2  [ **NUTF2** ]  exists in solution as dimers and, when bound to Sepharose beads, is able to interact specifically with both the Ras like GTPase  **Ran** , and the xFxFG repeat containing domains of nucleoporins p62 vertebrate and Nsp1p yeast.  Ref: 8918934 J Mol Biol, 1996
- Nuclear transport factor 2  [ **NUTF2** ]  NTF2  [ **NUTF2** ]  is associated with the translocation stage of nuclear protein import and binds both to nuclear pore proteins nucleoporins containing phenylalanine rich repeats and to the Ras family GTPase  **Ran** .  Ref: 9368653 J Mol Biol, 1997
- These results suggest that p10 NTF2  [ **NUTF2** ]  acts as a GDP dissociation inhibitor for  **Ran**   **Ran**  GDI, thereby coordinating the  **Ran**  dependent reactions that underlie nuclear protein import.  Ref: 9843686 Curr Biol, 1998
- Engineered mutants at the interaction interface reduce the binding of NTF2  [ **NUTF2** ]  to nuclear pores and cause reduced growth rates and  **Ran**  mislocalization when substituted for the wild type protein in yeast.  Ref: 12065398 EMBO J, 2002
- Furthermore, the activities of wild type p10 NTF2  [ **NUTF2** ]  and the mutant forms M84T and D92G in an assay of nuclear protein import in a digitonin permeabilized cell free system correlated with their level of inhibition of the dissociation of nucleotide from  **Ran**  GDP.  Ref: 9843686 Curr Biol, 1998

**Non-interactive relationship** (e.g. studied together, co-existance, homology, etc.)

- In contrast to NTF2  [ **NUTF2** ] , NXT1 preferentially binds  **Ran**  GTP, and it colocalizes with the nuclear pore complex NPC in mammalian cells.  Ref: 10567585 Mol Cell Biol, 1999
- Separate binding sites on nuclear transport factor 2  [ **NUTF2** ]  NTF2  [ **NUTF2** ]  for GDP  **Ran**  and the phenylalanine rich repeat regions of nucleoporins p62 and Nsp1p.  Ref: 8918934 J Mol Biol, 1996
- We conclude that NTF2  [ **NUTF2** ]  functions as a transport receptor for  **Ran** , permitting rapid entry into the nucleus where GTP GDP exchange mediated by RCC1 7 converts  **Ran**  into its GTP bound state.  Ref: 9889103 Curr Biol< MedlineTA>Curr Biol, 1996
- Nuclear import of proteins containing a nuclear localization signal NLS is dependent on the presence of a cytoplasmic NLS receptor, the GTPase  **Ran** , and p10 NTF2  [ **NUTF2** ] .  Ref: 9435235 Proc Natl Acad Sci U S A, 1998
- In this study, we show that mammalian p10 NTF2  [ **NUTF2** ]  dramatically inhibits the dissociation of 3H GDP from  **Ran**  and the binding of 35S GTPgammaS to  **Ran**  following the dissociation of non radioactive GDP by RCC1, the only known mammalian guanine nucleotide exchange factor for  **Ran**   **Ran**  GEF 7.  Ref: 9843686 Curr Biol, 1998
- The use of permeabilized cell models to study nuclear protein import has led to the identification of cytosolic components of the import machinery, including the NLS receptor, p97,  **Ran**  TC4  [ **RAN** ] , and nuclear transport factor 2  [ **NUTF2** ]  NTF2  [ **NUTF2** ] .  Ref: 8755535 Proc Natl Acad Sci U S A, 1996
- Recombinant importin alpha, importin beta,  **Ran** , and NTF2  [ **NUTF2** ]  alone were sufficient to support the import of both L form and D form conjugates in permeabilized cells.  Ref: 9792690 J Biol Chem, 1998
- This conformational change alters the positions of key residues such as Lys71, Phe72 and Arg76 that are crucial for the interaction of GDP  **Ran**  with NTF2  [ **NUTF2** ]  and indeed, solution binding studies were unable to detect any interaction between NTF2  [ **NUTF2** ]  and GDP RanQ69L under conditions where GDP  **Ran**  bound effectively.  Ref: 9878368 J Mol Biol, 1998
- When the structure of GDP RanQ69L from monoclinic crystals with P21 symmetry was compared with the structure of wild type  **Ran**  obtained from monoclinic crystals, the Q69L mutant showed a large conformational change in residues 68 74, which are in the switch II region of the molecule which changes conformation in response to nucleotide state and which forms the major interaction interface with nuclear transport factor 2  [ **NUTF2** ]  NTF2  [ **NUTF2** ] , sometimes called p10.  Ref: 9878368 J Mol Biol, 1998
- NLS dependent nuclear import was reconstituted in vitro using the different importin subunits together with the transport factors  **Ran**  and NTF2  [ **NUTF2** ] .  Ref: 10336485 J Biol Chem, 1999
- In the permeabilized cell in vitro transport system, nuclear import of SREBP 2 is reconstituted only by importin beta in conjunction with  **Ran**  and its interacting protein p10 NTF2  [ **NUTF2** ] .  Ref: 10397761 Mol Biol Cell, 1999
- These data suggest that certain of the nuclear protein import functions of NTF2  [ **NUTF2** ]  and  **Ran**  TC4  [ **RAN** ]  are closely linked.  Ref: 9261173 J Biol Chem, 1997
- Components necessary for translocation include the  **Ran**  TC4  [ **RAN** ]  GTPase and NTF2  [ **NUTF2** ]  B 2.  Ref: 8576188 J Biol Chem, 1996
- The cytosolic nuclear transport factor p10 NTF2  [ **NUTF2** ]  is required for the translocation of karyophilic molecules through nuclear pores 1 2 3, and the small GTPase  **Ran**  is a key regulator of protein transport between the nucleus and cytoplasm 4 5.  Ref: 9843686 Curr Biol, 1998
- Although the accumulation of NTF2  [ **NUTF2** ]  at the nuclear envelope is enhanced by RanGDP, we show that  **Ran**  binding does not alter the affinity of NTF2  [ **NUTF2** ]  for nucleoporins nor does the binding of nucleoporins alter the affinity of NTF2  [ **NUTF2** ]  for RanGDP.  Ref: 10681579 J Biol Chem, 2000
- Nuclear transport factor p10 NTF2  [ **NUTF2** ]  functions as a  **Ran**  GDP dissociation inhibitor  **Ran**  GDI.  Ref: 9843686 Curr Biol, 1998
- In contrast, a significant reduction in affinity of the RanGDP binding protein NTF2  [ **NUTF2** ]  for  **Ran**  CoreGDP is observed, indicating that the switch regions have changed conformation in the  **Ran**  Core mutant.  Ref: 12051861 J Mol Biol, 2002
- In rat liver nuclei, poly A RNA, hnRNPs A1 and C, mrnp 41, ASF, and a phosphorylated subset of SR proteins were also distributed like mRNA, as were various transport factors and their cargoes NTF2  [ **NUTF2** ] ,  **Ran** , RCC1, karyopherin beta, Rch1, transportin alpha, m, 7 trimethylG.  Ref: 10633080 J Cell Sci, 2000
- By preloading recombinant  **Ran**  TC4  [ **RAN** ]  with gamma 32P GTP or 3H GDP, we show that the interactions with p97 and NTF2  [ **NUTF2** ]  are specific for the GTP and GDP bound forms, respectively.  Ref: 8755535 Proc Natl Acad Sci U S A, 1996
- The structure of the Q69L mutant of GDP  **Ran**  shows a major conformational change in the switch II loop that accounts for its failure to bind nuclear transport factor 2  [ **NUTF2** ]  NTF2  [ **NUTF2** ] .  Ref: 9878368 J Mol Biol, 1998
- Surprisingly, we found that neither Xenopus ovarian cytosol nor a mixture of recombinant import factors karyopherin alpha2, karyopherin beta1,  **Ran** , and p10 NTF2  [ **NUTF2** ]  were able to support the import of pA RCC1 into the nuclei of digitonin permeabilized cells.  Ref: 10744690 J Biol Chem, 2000
- Nuclear protein accumulation in digitonin permeabilzed cells was impaired with  **Ran**  mutants deficient in NTF2  [ **NUTF2** ]  binding, confirming that the NTF2  [ **NUTF2** ]   **Ran**  interaction is required for efficient transport.  Ref: 10356329 J Mol Biol, 1999
- We have used a range of complementary biochemical and biophysical methods to investigate the interactions between nuclear transport factor 2  [ **NUTF2** ]  NTF2  [ **NUTF2** ] , the Ras family GTPase  **Ran** , and XFXFG nucleoporin repeats that are crucial for nuclear trafficking.  Ref: 10681579 J Biol Chem, 2000
- The precise role s of p10 NTF2  [ **NUTF2** ]  in the  **Ran**  GTP GDP cycle are thus far unclear, however.  Ref: 9843686 Curr Biol, 1998
- Here, we examine the in vivo role of NTF2  [ **NUTF2** ]  in  **Ran**  import and the effect that disruption of  **Ran**  imported into the nucleus has on the cell cycle.  Ref: 10930458 Mol Biol Cell, 2000
- The aromatic side chain of  **Ran**  Phe72 inserts into the NTF2  [ **NUTF2** ]  cavity and accounts for 22% of the surface area buried by the interaction interface, while salt bridges are formed between Lys71 and Arg76 of  **Ran**  with Asp92 Asp94 and Glu42 of NTF2  [ **NUTF2** ] , respectively.  Ref: 9533885 J Mol Biol, 1998
- The TAP p15 heterodimer is structurally similar to the homodimeric transport factor NTF2  [ **NUTF2** ] , but unlike NTF2  [ **NUTF2** ] , it is incompatible with either homodimerization or  **Ran**  binding.  Ref: 11583626 Mol Cell, 2001
- We have used X ray crystallography to determine the structure of the macromolecular complex formed between GDP  **Ran**  and nuclear transport factor 2  [ **NUTF2** ]  NTF2  [ **NUTF2** ]  at 2.5 A resolution.  Ref: 9533885 J Mol Biol, 1998
- Four mutant complementation groups were characterized that correspond to respective mutations in genes encoding  **Ran**  gsp1, and essential  **Ran**  regulatory factors  **Ran**  GTPase  [ **RAN** ]  activating protein rna1,  **Ran**  guanine nucleotide exchange factor prp20, and the RanGDP import factor ntf2  [ **NUTF2** ] .  Ref: 12654904 J Cell Biol, 2003
- Consistent with a critical role for NTF2  [ **NUTF2** ]  in establishing and maintaining the  **Ran**  gradient, we show that NTF2  [ **NUTF2** ]  is required for early embryogenesis in Caenorhabditis elegans.  Ref: 10889207 J Biol Chem, 2000
- **Ran**  and p62 do not compete with one another for binding to NTF2  [ **NUTF2** ] , indicating that they bind to different sites on NTF2  [ **NUTF2** ] .  Ref: 8918934 J Mol Biol, 1996
- The GDP bound form of the switch II mutants showed no detectable binding to NTF2  [ **NUTF2** ] , providing direct evidence that salt bridges involving Lys71 and Arg76 and burying Phe72 are all crucial for the interaction between  **Ran**  and NTF2  [ **NUTF2** ] .  Ref: 10356329 J Mol Biol, 1999
- Purified mutant proteins retained the interaction with FxFG repeat nucleoporins, but several mutants in the negatively charged residues that surround the NTF2  [ **NUTF2** ]  cavity or in residues in the cavity itself were unable to bind GDP  **Ran**  in vitro.  Ref: 9368653 J Mol Biol, 1997
- ... addition of transportin, which accumulates in the nucleus, enhanced NTF2  [ **NUTF2** ]  dependent  **Ran**  import.  Ref: 9889103 Curr Biol< MedlineTA>Curr Biol, 1997
- To analyze the mechanism of  **Ran**  import into the nucleus by NTF2  [ **NUTF2** ] , we use mutants in a variety of nuclear transport factors along with biochemical analyses of NTF2  [ **NUTF2** ]  complexes.  Ref: 10889207 J Biol Chem, 2000
- In contrast, the dissociation of 35S GTP gamma S from  **Ran** , which was also catalyzed by RCC1, was not affected by p10 NTF2  [ **NUTF2** ] .  Ref: 9843686 Curr Biol, 1998
- Nuclear protein import is decreased by engineered mutants of nuclear transport factor 2  [ **NUTF2** ]  NTF2  [ **NUTF2** ]  that do not bind GDP  **Ran** .  Ref: 9368653 J Mol Biol, 1997
- NTF2  [ **NUTF2** ]  mediated  **Ran**  nuclear import is required for NLS dependent protein import.  Ref: 10679025 Mol Biol Cell, 2000
- We find that  **Ran**  remains concentrated in the nucleus when importin mediated protein import is disrupted and demonstrate that NTF2  [ **NUTF2** ]  does not form a stable complex with the transport receptor, importin beta.  Ref: 10889207 J Biol Chem, 2000
- The GTPase  **Ran**  TC4  [ **RAN** ]  and the 14 kDa protein nuclear transport factor 2  [ **NUTF2** ]  NTF2  [ **NUTF2** ]  are two of the cytosolic factors that mediate nuclear protein import in vertebrates.  Ref: 9261173 J Biol Chem, 1997
- In this study we probed the role of the NTF2  [ **NUTF2** ]   **Ran**  interaction in nuclear protein import using site directed mutants of NTF2  [ **NUTF2** ]  that interfere with its interaction with GDP  **Ran** .  Ref: 9368653 J Mol Biol, 1997
- The mechanism of  **ran**  import into the nucleus by nuclear transport factor 2  [ **NUTF2** ] .  Ref: 10889207 J Biol Chem, 2000
- Our data distinguish between two possible mechanisms for  **Ran**  import by NTF2  [ **NUTF2** ]  and demonstrate that  **Ran**  import is independent from importin beta mediated protein import.  Ref: 10889207 J Biol Chem, 2000
- In permeabilised cell nuclear protein import assays, only wild type NTF2  [ **NUTF2** ]  and mutants that bound GDP  **Ran**  were functional.  Ref: 9368653 J Mol Biol, 1997
- the association of the GDP bound form of  **Ran**  TC4  [ **RAN** ]  with NTF2  [ **NUTF2** ]  helps define vectorial transport.  Ref: 8755535 Proc Natl Acad Sci U S A, 1996
- The second step, translocation, requires at least two soluble factors, a small GTPase  **Ran**  and its interacting protein p10 nuclear transport factor 2  [ **NUTF2** ]  NTF2  [ **NUTF2** ] , along with nuclear pore complex components.  Ref: 9192717 J Biochem Tokyo , 1997
- Nuclear transport factor 2  [ **NUTF2** ]  NTF2  [ **NUTF2** ]  and the Ras family GTPase  **Ran**  are two soluble components of the nuclear protein import machinery.  Ref: 9533885 J Mol Biol, 1998
- The binding of GDP  **Ran**  at the NTF2  [ **NUTF2** ]  cavity raises the possibility that this interaction might be modulated by a metabolite or small molecule substrate for NTF2  [ **NUTF2** ]  s putative enzymatic activity.  Ref: 9533885 J Mol Biol, 1998
- 4 Importin beta1,  **Ran** , and p10 NTF2  [ **NUTF2** ]  are sufficient to mediate import of activated Smad3.  Ref: 11294908 Mol Biol Cell, 2001
